# Intrauterine Perfusion of Autologous Platelet-Rich Plasma Before Frozen-Thawed Embryo Transfer Improves the Clinical Pregnancy Rate of Women With Recurrent Implantation Failure

**DOI:** 10.3389/fmed.2022.850002

**Published:** 2022-03-29

**Authors:** Yangying Xu, Cuifang Hao, Jianye Fang, Xiaoqiang Liu, Pingping Xue, Ruichao Miao

**Affiliations:** Qingdao Women and Children's Hospital Affiliated to Qingdao University, Qingdao, China

**Keywords:** platelet-rich plasma (PRP), frozen-thawed embryo transfer (FET), intrauterine perfusion, live birth rate, clinical pregnancy rate (CPR)

## Abstract

**Objective:**

To evaluate whether the intrauterine perfusion of platelet-rich plasma (PRP) before frozen-thawed embryo transfer (FET) improves the pregnancy outcomes of patients with repeated implantation failure (RIF).

**Methods:**

This retrospective study included 288 infertile women with RIF after undergoing *in vitro* fertilization/intracytoplasmic sperm injection (IVF/ICSI) treatment from October 1, 2019, to January 1, 2021, at Qingdao Women and Children's Hospital. Patients were divided into two groups according to whether they received PRP intrauterine perfusion before embryo transfer in FET cycles. 138 women were in the PRP group, 150 women were in the control group. The primary outcome measure was live birth rates and the secondary outcome were clinical pregnancy, positive β hCG, miscarriage and implantation rates.

**Results:**

No significant differences in baseline demographic and clinical characteristics were observed between the two groups. Overall, significantly more women in the PRP group than in the control group achieved a live birth rate (41 women; 29.71% vs. 27 women; 18%) and a clinical pregnancy (50 women; 36.23% vs. 37 women; 24.67%). The PRP group had a higher implantation rate and lower spontaneous miscarriage rate than the control group, but these differences were not statistically significant. No pregnancy outcome difference between two groups in PCOS patients with RIF.

**Conclusion:**

Our results showed that intrauterine perfusion of PRP before embryo transfer in FET cycles can significantly increase the live birth and clinical pregnancy rates in patients with RIF.

## Introduction

In the context of *in vitro* fertilization-embryo transfer (IVF-ET), RIF refers to patients for whom implantation fails after repeated transfers of morphologically good-quality embryos into a normal uterus (1). Despite great progress in the application of IVF-ET to infertile couples, recurrent implantation failure (RIF) following embryo transfer (ET) continues to be a major problem affecting about 10% of women causing great psychological and economic burden to individuals and their families (2, 3), New strategies to improve the pregnancy likelihoods are urgently needed for these patients.

The successful implantation depends on embryo quality and endometrial receptivity. Endometrial receptivity is a complex process that involves the participation of and regulation by many molecules, including cytokines, growth factors, adhesion molecules, and cytoskeletal proteins, among other factors (4). Patients with RIF are in most due to endometrial receptivity disorders. numerous studies have investigated ways to improve endometrial receptivity, and the methods examined have included low-dose aspirin therapy, intrauterine infusion of human chorionic gonadotrophin (HCG) or colony cell-stimulating factor (CSF), endometrial injury and so like (5–8), however, the optimal technique remains unclear.

PRP is an autologous blood-derived concentrate of platelets from peripheral blood with a considerable concentration of growth factors and cytokines. Since its introduction in 1970, PRP has been widely used as a form of regenerative treatment in multiple medical fields, such as sports medicine, maxillofacial surgery and cosmetology. Studies in recent years have shown great promise in reproduction fields. Many studies have demonstrated that autologous PRP can regenerate and thicken the thin endometrium and decrease the likelihood of implantation failure leading to good pregnancy outcomes (9–11), however the patient's heterogeneity and small sample size of the subjects led to different research results, therefore this research analyzed the use of PRP before embryo transfer in RIF patients of frozen thawed cycles and its efficacy in this population.

## Materials and Methods

### Study Design

This retrospective study was carried at reproductive center of Qingdao Women and Children's Hospital. We retrospectively analyzed the records of 410 patients with history of RIF underwent FET in Qingdao Women and Children's Hospital from October 1, 2019, to January 1, 2021. 138 patients (screened from 168 patients) who received PRP intrauterine perfusion before embryo transfer in frozen thawed cycles were collected, 150 patients (screened from 242 patients) with history of RIF did not receive PRP intrauterine perfusion as the control group. We excluded 122 patients who did not meet the criteria and had incomplete data. No repeat treatment cycle for all patients. Clinicians and this PRP intrauterine treatment study were authorized by the ethics committee of Qingdao Women and Children's Hospital. Informed consent was obtained from all patients with PRP intrauterine perfusion on endometrial transformation day, follow-up was complete in all patients.

### Patient Enrolment Criteria

Patients aged 23 to 40 years who had three or more consecutive failed embryo implantations with good-quality embryos (at least 6 cleavage-stage embryos or three blastocysts) were included in the study (12). The exclusion criteria were an abnormal karyotype from either partner, evidence of uterine defects, ultrasonographic evidence of hydrosalpinx, infections, endocrine problems, coagulation defects or autoimmune defects.

Endometrial preparation for frozen-thawed cycle included hormone replacement treatment (HRT) protocol (estrogen-progesterone cycle, EP) and natural cycle (NC). PRP intrauterine perfusion was performed 48–72 h before embryo transfer. One or two good quality embryos were transferred. The rates of live birth, clinical pregnancy, positive-hCG, miscarriages and implantation will be assessed. Serum β-hCG was measured *14* days after embryo transfer and was considered positive for β- hCG level ≥ 5IU. Clinical pregnancy was confirmed by transvaginal ultrasonography 30 days after embryo transfer.

### Plate-Rich Plasma Preparation and Intrauterine Perfusion

Twenty milliliters of peripheral blood were collected from each patient and centrifuged at 500 × g for 10 min at 18°C. After centrifugation, three layers were visible, namely, the serum, platelets and red blood cells. The serum and platelets were transferred to another tube and centrifuged at 500 × g for another 10 min at 18°C. The supernatant was discarded, the remaining 1 mL PRP was infused into the uterine cavity using artificial insemination catheter 2 days before embryo transfer (13). The mean number of platelets in PRP was 1513.45 ± 322.18 × 10^9^/L (Automated Hematology Analyzer sysmex xs-500i, Sysmex Corporation, Kobe, Japan). All perfusions were prepared and performed by a single operator.

### Statistical Analysis

All statistical analyses were performed using SPSS 22.0 software (IBM Corp., Armonk, NY, USA). Student's *t*-test was used for comparisons of continuous variables between the groups. The chi-squared test or Fisher's exact test, where appropriate, was used for comparisons of categorical variables. The results are presented as the mean ± standard deviation (SD). Statistical significance was set at a probability (*P*) value < 0.05.

## Results

A total of 288 patients with an RIF history were included in this study; 138 patients undergoing intrauterine PRP perfusion before embryo transfer in frozen-thawed cycles were included in the PRP group, and the remaining 150 women who were not undergoing PRP perfusion were included in the control group. The mean age was 35.7 ± 4.6 years, the mean duration of infertility was 7.8 ± 3.7 years and the mean number of history embryo transfer cycles was 3.72 ± 1.09 and the control was 3.67 ± 0.98. No local or systemic side effects relating to the use of PRP perfusion were reported (Table 1).

**Table 1:** Baseline characteristics of the participants with RIF patients.

	**PRP group** **(*n* = 138)**	**Control group** **(*n* = 150)**	***P*-value**
**Demographic characteristics**			
Female age (years), mean ± SD	34.92 ± 4.80	34.93 ± 4.87	0.986
Male age (years), mean ± SD	35.83 ± 5.72	36.71 ± 6.65	0.232
BMI (kg/m^2^), mean ± SD	24.08 ± 3.65	24.81 ± 3.90	0.103
FSH (IU/ml), mean ± SD	6.69 ± 1.50	6.45 ± 1.47	0.172
AMH (ng/ml), mean ± SD	2.38 ± 2.45	2.40 ± 3.03	0.951
Infertility duration, mean ± SD	4.35 ± 3.29	4.28 ± 3.74	0.867
Number of history ET cycles	3.72 ± 1.09	3.67 ± 0.98	0.682
**Type of infertility**			
Primary infertility, *n* (%)	75/138 (54.35)	78/150	0.159
Secondary infertility, *n* (%)	63/138 (45.65)	72/150	0.159
**Diagnosis of infertility**			
Tubal factor, *n* (%)	44/138 (31.89)	49/150	0.821
Male factor, *n* (%)	41/138 (29.71)	44/150	0.944
PCOS, *n* (%)	29/138 (21.01)	38/150	0.386
Other causes, *n* (%)	20/138 (14.49)	19/150	0.219

*The values are expressed as the mean ± SD*.

Regarding the patients' baseline demographic characteristics, we found no differences between the two groups in age, body mass index (BMI), duration of infertility, number of history cycles transferred or infertility diagnosis between the two groups. There were also no differences in cycle characteristics, such as endometrial thickness, number of days embryo transfer, or number of blastocysts or cleavage-stage embryos transferred between the two groups (Table 2).

**Table 2:** Clinical characteristics of the participants with RIF patients.

	**PRP group** **(*n* = 138)**	**Control group** **(*n* = 150)**	***P*-value**
Endometrial thickness, mean ± SD (Endometrial conversion day)	980 ± 2.27	10.06 ± 2.16	0.320
Number of embryo transfers, mean ± SD	1.48 ± 0.50	1.40 ± 0.50	0.176
Number of blastocysts, *n* (%)	60/138 (43.48)	62/150 (41.33)	0.713
Number of cleavage-stage embryos, n (%)	78/138 (57.78)	88/150 (58.67)	0.713
Positive β hCG rates, *n* (%)	62/138 (44.93)^*^	45/150 (30.00%)	<0.01^*^
Implantation rate, *n* (%)	56/206 (27.18)	37/210 (17.62)	0.067
Biochemical pregnancies, *n* (%)	12/138 (8.70)	8/150 (6.67)	0.156
Clinical pregnancies, *n* (%)	50/138 (36.23)^*^	37/150 (24.67)	0.040^*^
Spontaneous miscarriages, *n* (%)	9/50 (18.00)	10/37 (27.03)	0.454
Live births, *n* (%)	41/138 (29.71)^*^	27/150 (14)	<0.01^*^

* *Indicate significant differences between the two groups*.

To evaluate whether PRP improves the reproductive outcomes of patients with RIF, we first calculated the live birth rates and the clinical pregnancy rates in two groups. In our study, there were 41 live birth in the PRP intrauterine perfusion group (41/138) and 27 in the control group (27/150), the live birth rate was significantly higher in the PRP group (29.71%) than in the control group 18% (*P* < 0.01). Additionally, the clinical pregnancy rate was also higher in the PRP group 36.23% than in the control group 24.67% (*P* = 0.040). Further analysis of the secondary outcomes showed that the Positive β hCG rates is significantly higher in PRP group 44.93% (62/138) than the control group 28.66% (*P* < 0.01). None of other secondary outcome indices were significantly different between the two groups, that the implantation rates were 27.18% and 17.62% (*P* = 0.067), respectively; that the biochemical pregnancy rates were 7.97% and 6.675 (*P* = 0.156), respectively; and that the miscarriage rates were 18.0% and 27.03% (*P* = 0.454), respectively (Table 2). There is also no in clinical pregnancy and live birth rate difference between PRP group and control group in RIF PCOS patients (Table 2).

## Discussion

This retrospective cohort study was performed to evaluate the pregnancy outcomes of RIF patients who underwent PRP intrauterine perfusion before embryo transfer and compare these results to a cohort of RIF patients who were not treated with PRP. We found there is significant different in live birth and clinical pregnancy rates, we have also found there is significantly different in positive β-hCG rates, no significantly difference were found in the implantation rates and miscarriages.

Yajie Chang, Sunita R, Tandulwadkar, and Eftekhar have demonstrated that autologous PRP intrauterine perfusion could expanded the thin endometrium and increase the clinical pregnancy and implantation rates in FET cycles (15–17), they think that PRP could regenerate and improve the endometrium receptivity leading to good pregnancy outcomes. Hakan Coksuer also found that PRP intrauterine perfusion could thicken the endometrium leading to good endometrial lining and optimal pregnancy outcomes in RIF patients (18). Xiaohan Wang and colleagues found that PRP significantly stimulated the growth, migration and adhesion of endometrial mesenchymal stem cells, these effects are probably because it contains or stimulates several growth factors such as transforming growth factor, Platelet-derived growth factor, vascular endothelial growth factor, insulin like growth factor, epithelial growth factor and bioactive-cytokines (14). However, these majority of studies had small sample cases, most less than 50 patients and almost all fewer than 100 patients. the live birth rate was not reported in most studies. Our study is relatively larger sample size of 238 patients, we found no difference in endometrium thickness between groups, the live birth rate, clinical pregnancy, positive β-HCG rates are higher than the control group, the results are consistent with the literature. We think those biological factors may acts in most by improving and triggering endometrial receptivity through the improvement of cell proliferation, vascularization, anti-inflammatory properties, rather than expanded endometrial growth.

However, contradictory views on the usefulness of PRP perfusion treatment in IVF reproductive medicine persists. Tehraninejad and Allahveisi found that the pregnancy outcomes, namely, the clinical and ongoing pregnancy rates, live birth rates were similar between the PRP intrauterine infusion group and the control group in RIF patients of FET cycles (10, 11). The reason for the inconsistency between our results and those of others might be attributable to the different subject cohorts, different definitions of RIF and wide variety of *PRP* preparation protocols. The PRP in our study contains leukocytes in addition to the platelets and plasma, it is categorized as leucocyte-poor PRP, not pure PRP. leukocytes might be also a source of several growth factors and cytokines (19). The concentrations of platelets and leukocytes obtained in PRP preparation depending on how the blood sample was treated, which may in turn impact the amount of various types of growth factors playing a key part in the treatment.

To date, only a few RCT studies on intrauterine infusion of PRP have been performed in the reproductive field. Eftekhar and Nazari suggested that PRP could expand the thin endometrium and improve clinical pregnancy outcomes (17, 20), while Allahveisi's study concluded that intrauterine infusion of PRP did not improve pregnancy outcomes in patients with recurrent implantation failure (11). Design of placebo in the control group was inconsistent across studies. The intrauterine perfusion of PRP is associated with the potential effect of the endometrial injury, is it possible the beneficial effect is due to the endometrial injury of the catheter? So we should design studies carefully, especially RCTs, larger scales, the standard PRP preparation procedure and the strict indication of the PRP treatment should be all considered.

Another notable point is that PRP intrauterine perfusion did not improve pregnancy outcomes in RIF PCOS patients. There is no significant difference between the two groups. This might be caused by the small sample size in this study. Besides, PCOS is a special group where obesity, insulin resistance, abnormal glucose metabolism and metabolic syndrome may alter the competence of oocytes, embryos and endometrium (21). Palomba's comprehensive review shows that among several interventions to improve endometrial receptivity in women with PCOS, only lifestyle modification, metformin and bariatric surgery have the scientific evidence for clinical benefit (22).

The strength of this study is that we included a large sample of RIF patients with the PRP intervention before embryo transfer in our reproductive center, which allows us to draw a conclusion on the efficacy of this strategy. The main drawback of this study was retrospective and its non-randomized design. The findings provide evidence for future randomized, controlled trials with large sample size in this field. One further prospective and high-quality randomized controlled trial is ongoing in our center to identify subpopulations that would benefit most from PRP therapy.

In summary, we analyzed the effect of intrauterine PRP treatment before embryo transfer in patients with RIF and found that the PRP intervention improved the live birth rate and clinical pregnancy rate.

## Data Availability Statement

The original contributions presented in the study are included in the article/supplementary material, further inquiries can be directed to the corresponding author.

## Ethics Statement

The studies involving human participants were reviewed and approved by Ethics Committee of Qingdao Women and Children's Hospital. The patients/participants provided their written informed consent to participate in this study.

## Author Contributions

YX took part in the first draft and preparation of the manuscript. JF, XL, PX, and RM collected material and data. CH contributed to the concept, design and preparation of the manuscript. All authors were involved in the study conception and design and read and approved the final manuscript.

## Funding

This study was supported by Qingdao Key Health Discipline Development Fund.

## Conflict of Interest

The authors declare that the research was conducted in the absence of any commercial or financial relationships that could be construed as a potential conflict of interest.

## Publisher's Note

All claims expressed in this article are solely those of the authors and do not necessarily represent those of their affiliated organizations, or those of the publisher, the editors and the reviewers. Any product that may be evaluated in this article, or claim that may be made by its manufacturer, is not guaranteed or endorsed by the publisher.
